# Early Prediction of Cardiogenic Shock Using Machine Learning

**DOI:** 10.3389/fcvm.2022.862424

**Published:** 2022-07-13

**Authors:** Yale Chang, Corneliu Antonescu, Shreyas Ravindranath, Junzi Dong, Mingyu Lu, Francesco Vicario, Lisa Wondrely, Pam Thompson, Dennis Swearingen, Deepak Acharya

**Affiliations:** ^1^Philips Research North America, Cambridge, MA, United States; ^2^Division of Cardiovascular Disease, Banner Health, Tucson, AZ, United States; ^3^University of Arizona College of Medicine, Phoenix, AZ, United States; ^4^Department of Computer Science, University of Washington, Seattle, WA, United States

**Keywords:** cardiogenic shock, machine learning, electronic health records, early warning system, clinical decision support, subpopulation analysis

## Abstract

Cardiogenic shock (CS) is a severe condition with in-hospital mortality of up to 50%. Patients who develop CS may have previous cardiac history, but that may not always be the case, adding to the challenges in optimally identifying and managing these patients. Patients may present to a medical facility with CS or develop CS while in the emergency department (ED), in a general inpatient ward (WARD) or in the critical care unit (CC). While different clinical pathways for management exist once CS is recognized, there are challenges in identifying the patients in a timely manner, in all settings, in a timeframe that will allow proper management. We therefore developed and evaluated retrospectively a machine learning model based on the XGBoost (XGB) algorithm which runs automatically on patient data from the electronic health record (EHR). The algorithm was trained on 8 years of de-identified data (from 2010 to 2017) collected from a large regional healthcare system. The input variables include demographics, vital signs, laboratory values, some orders, and specific pre-existing diagnoses. The model was designed to make predictions 2 h prior to the need of first CS intervention (inotrope, vasopressor, or mechanical circulatory support). The algorithm achieves an overall area under curve (AUC) of 0.87 (0.81 in CC, 0.84 in ED, 0.97 in WARD), which is considered useful for clinical use. The algorithm can be refined based on specific elements defining patient subpopulations, for example presence of acute myocardial infarction (AMI) or congestive heart failure (CHF), further increasing its precision when a patient has these conditions. The top-contributing risk factors learned by the model are consistent with existing clinical findings. Our conclusion is that a useful machine learning model can be used to predict the development of CS. This manuscript describes the main steps of the development process and our results.

## 1. Introduction

CS is a condition characterized by low cardiac output leading to hypoperfusion of major organs and is associated with a high short-term mortality of up to 50% (1). Clinical trials of CS patients are difficult to conduct because of high patient acuity, limited time for therapeutic interventions, and heterogeneity of CS. As a result, limited data exists regarding efficacy of various therapeutic interventions, and major gaps are present in the understanding of the most appropriate therapy in individual patients at various stages of disease. Studies have evaluated the predictors of mortality in patients who are diagnosed with CS (2). However, these predictors are generally non-modifiable and are assessed after the patient has already developed CS. While they are able to predict clinical outcomes, they typically fail to provide adequate information in a manner to influences patient management and therefore increase chances of improve survival. Several studies have also evaluated predictors of developing CS, but these are usually static variables or data available post intervention (e.g., thrombolysis in MI flow) that don't provide early discrimination (3).

Early recognition, triage, risk stratification and protocolized management of patients in hospitals equipped with adequately trained personnel and technology have been associated with improved outcomes in observational studies of CS (4). Many patients who experience CS may not present initially to tertiary care hospitals but rather to smaller hospitals without specialized cardiovascular or CS therapies available. Furthermore, a substantial proportion don't present to the hospital in CS but develop it after they have been admitted to the hospital for other reasons, such as AMI or CHF (5, 6). They may also develop CS during non-working hours when there are fewer clinicians at bedside who are capable of immediately evaluating, recognizing, appropriately triaging, managing, and potentially transferring patients to specialized centers. Thus, there exists an important unmet need for methods to consistently identify patients who are at risk of developing CS.

The widespread use of EHR systems enables development of algorithms to identify specific patient parameters, abnormalities, and provide individualized decision support. Machine learning algorithms can perform automated, continuous screening and therefore be integrated into standard clinical workflows to provide early notification. The purpose of this study is to develop an early warning system for CS using machine learning models and based on routinely populated clinical variables available from the EHR within a large regional healthcare system.

Specifically, our objective is to predict the development of CS 2 h earlier than with standard clinical care. In this way, clinicians can reassess the patient's condition and provide early cardiac interventions to prevent further deterioration into CS. To the best of our knowledge, our work presents the first model that can predict the onset of CS for the general patient population with good performance.

We chose to use machine learning techniques because they often achieve significantly better prediction performance compared to standard statistical models such as logistic regression (LR) (7). Machine learning algorithms can (1) model non-linear relationships between input variables and the target variable; (2) naturally incorporate interactions between different inputs; (3) achieve interpretability. We evaluate multiple machine learning algorithms as well as LR and choose the algorithm [XGB, (8)] achieving the best prediction performance.

## 2. Materials and Methods

In this section, we first describe the extraction process of the patient cohort, which consists of 4,012 CS patients, 782 hypovolemic shock patients, 16,916 septic shock patients, and 93,581 non-shock patients from the EHR data of a large regional healthcare system. For each patient, 76 clinical variables, including vital signs, laboratory measurements, ventilator settings, previous diagnoses and interventions, were extracted.

Then we describe the labeling of positive and negative class used for model training. The XGB classifier was chosen to build the CS prediction model because it achieved the optimal performance. Compared to LR, where the log-odds is assumed to be the linear weighted summation of input variables, XGB assumed the log-odds as the summation of hundreds or even thousands of decision trees applied to input variables. Since each decision tree defines a non-linear function over multiple input variables, such as predicting the positive class if systolic blood pressure falls below 90 bpm and body temperature is below 36 degrees Celsius, XGB automatically learns non-linear combinations of input variables and can identify more input patterns that are predictive of the target variable compared to LR.

### 2.1. Patient Cohort Extraction

We extracted a patient cohort consisting of (1) patients with diagnosis of CS based on ICD code which we used as target population; and (2) patients without diagnosis of CS as control, from a large-scale longitudinal patient EHR database collected from Banner Health, which is a large regional healthcare system consisting of 30 hospitals in the US, from 2010 to 2017. The use of the patient data was approved by the Institutional Review Board. In this database, more than 11 million patients have measurements of systolic blood pressure during their stays.

#### 2.1.1. Target Patients With CS

Both ICD-9 and ICD-10 codes of CS, including R57.0, 785.51, 998.01, were used to identify the target patients. In total, 5,881 target patients were identified.

For each target patient, the onset time of CS was determined as follows: we used the onset time of the first clinical interventions typically given to CS patients, including vasopressors, inotropes, or mechanical circulatory support, as the surrogate of the onset time of CS. Patients who received ICD diagnosis of CS but no CS-specific interventions were excluded. Since patients often received a sequence of interventions over time, the onset time of the first intervention was selected to determine the onset time of CS. The list of interventions used are summarized in Table 1.

**Table 1:** Clinical interventions often applied to patients with CS.

Vasopressors	Norepinephrine, Epinephrine, Dopamine, Phenylephrine, Vasopressin
Inotropes	Dobutamine, Milrinone
Mechanical cardiac support	IABP, Impella, LVAD, ECMO, TandemHeart

We carefully assessed but ultimately did not use a blood pressure threshold in isolation to determine the onset of CS for multiple reasons: (1) a single low blood pressure measurement may be erroneous or may not provide enough specificity for the onset of CS, especially if the subsequent reading is in normal physiological range without intervention; (2) a single low blood pressure measurement in the absence of ancillary clinical evidence of hypoperfusion may not indicate onset of CS. For example, many advanced heart failure patients who eventually develop CS may have baseline hypotension; (3) hypotension may be caused by other causes including hypovolemia, medications, arrhythmia, and septic shock rather than onset of CS. We also evaluated using two consecutive blood pressure readings indicative of hypotension to determine the onset of CS. However, the limitations of this method were that there were often large gaps in time between the two low measurements, precluding accurate determination of true of timing of shock. To consider an example case scenario, patients who were transferred to operating room (OR) or catheterization laboratory (Cath lab) after shock had hemodynamic measurements, including blood pressure, recorded in the OR/Cath lab recording software, which was not readily available in the EHR, leading to large gaps in the measurements extractable from the EHR. In another scenario, in patients who had continuous hemodynamic monitoring (e.g., arterial lines), not all hypotensive episodes would be recorded in the EHR. Furthermore, patients who had shock and were treated quickly with vasopressors/inotropic agents may not have had multiple hypotensive measurements despite true CS. For these reasons, we opted to choose the time of recognition and institution of therapy in current practice as the onset time of CS and aimed to improve upon this.

Among 5,881 CS patients identified through the ICD codes listed above, 5316 patients (90%) received at least one type of interventions listed in Table 1. For each patient who received interventions, we identified the type of the first intervention and computed their frequency for all such patients as shown in Table 2. Note that the total number of patients shown in the second column exceeds 5316 because some patients received multiple types of interventions at the same time.

**Table 2:** The distribution of the type of first intervention received by patients with diagnosis of CS.

**Type of first intervention**	**Number of patients**	**Percentage**
		**(%)**
Norepinephrine	2,524	47
Dopamine	1,057	20
Dobutamine	691	13
Epinephrine	642	12
IABP	458	9
Phenylephrine	430	8
Milrinone	385	7
Vasopressin	214	4
VAD	92	2
Impella	70	1
ECMO	18	0

Norepinephrine, Dopamine, Dobutamine, and Epinephrine were first-line agents. Second, compared to Norepinephrine, other pressors were much less likely to be used first. Third, mechanical support devices were rarely applied as first line interventions since they take time to arrange and are usually applied after pressors/inotropes are administered. These findings were consistent with the current practices of treating CS patients (9). We further restricted the target patient cohort to only include adult patients (age greater than 18 years), reducing the cohort size from 5,316 to 5,148.

#### 2.1.2. Control Patients

The control patients consisted of both shock patients and non-shock patients. Specifically, for shock patients, we extracted all patients with diagnosis of either septic shock or hypovolemic shock. Ideally, the trained model should separate CS from other types of shock. Both septic shock and hypovolemic shock patients were identified using ICD codes. Their onset time was determined by the first administration of vasopressors, including Norepinephrine, Epinephrine, Dopamine, Phenylephrine, and Vasopressin. For non-shock patients, we randomly sampled 100,000 patients from the entire patient cohort after excluding shock patients (around 11 million patients). Although the model automatically balanced the control cohort and the target cohort, the subsampling was needed to avoid creating a patient cohort that is too large to apply standard analytics tools.

After excluding non-adult patients, the number of patients belonging to the septic shock, hypovolemic shock and non-shock groups are shown in Table 3.

**Table 3:** Patients with septic shock, hypovolemic shock, and no shock in the control patient group.

**Subset of control patients**	**Number of patients**
Septic shock	18,321
Hypovolemic shock	1,171
Non-shock	93,581

#### 2.1.3. Combine Target Patients and Control Patients

In practice, one patient can develop multiple types of shock. For example, CS patients can develop septic shock during their stay in the intensive care units. To train a model that can separate CS from other shock types, we only kept patients with diagnosis of a single type of shock due to the lack of information to determine with certainty what type of shock occurred first. Since patients with diagnosis of multiple shock types were excluded, the number of patients in both the target group and the control group further decreased (Table 4).

**Table 4:** Number of patients in the target group (CS) and the control group (septic/hypovolemic shock and non-shock) after excluding patients with diagnosis of multiple shock types.

**Patient group**		**Number of patients**	**Percentage**
			**(%)**
Target	CS	4,012	3.5
Control	Hypovolemic shock	782	0.7
Control	Septic shock	16,916	14.7
Control	Non-shock	93,581	81.1

#### 2.1.4. Input Feature Extraction

We extracted 76 variables, including vital signs, laboratory measurements, ventilator settings, previous diagnoses, antibiotics administration and echocardiogram. The names of these variables are listed in Table 5. The naming of most vital signs, laboratory measurements and ventilator settings are self-explanatory. For variables that need further explanation, detailed descriptions are in Section 1 in the Supplementary Material.

**Table 5:** Input variables extracted for mode training.

ALT	CVP	Hemoglobin	PlateletCount
AMI	Calcium	INR	Potassium
AST	Carboxyhemoglobin	ImmatureGranulocytes	Procalcitonin
AVPU_Scale	CardiacIndex	Lactate	RBC
AgeInYears	Cardiomyopathy	Lymphocytes	Respiration
Albumin	Chloride	Magnesium	SAFE_SIRS
AnionGap	Compliance	MeanPlateletVolume	Sodium
Antibiotics	Creatinine	Methemoglobin	SystolicBloodPressure
BUN	D_Dimer	MitralRegurgitation	PulsePressure
Bands	ECHO	NT-proBNP	Temperature
BaseExcess	Eosinophils	Neutrophils	Troponin
Basophils	EWS_CNS	O2Saturation	TroponinDelta
Bicarbonate	EjectionFraction	PAWP	WBC
Bilirubin	Fio2	PCO2	AirwayPressure
BloodCulture	GFR	PEEP	InspiratoryTime
CKMB	GenderIsMale	PIP	TidalVolume
CKMB_CKTotal	Glucose	PO2	MinuteVolume
CKTotal	HeartRate	PT	pH
CRP	Hematocrit	PTT	PlateauPressure


##### 2.1.4.1. Time Range of Input Features

For each patient, starting from the time of intervention, we extracted the input variable values from 1 to 12 h before the intervention onset time. Note that the time of intervention for non-shock patients was randomly selected in their hospital stay. At each time point, the value measured in the last 2 h was used for vital signs or 48 h for laboratory measurements or ventilator settings.

#### 2.1.5. Patient Cohort Statistics

**Figure 1:**
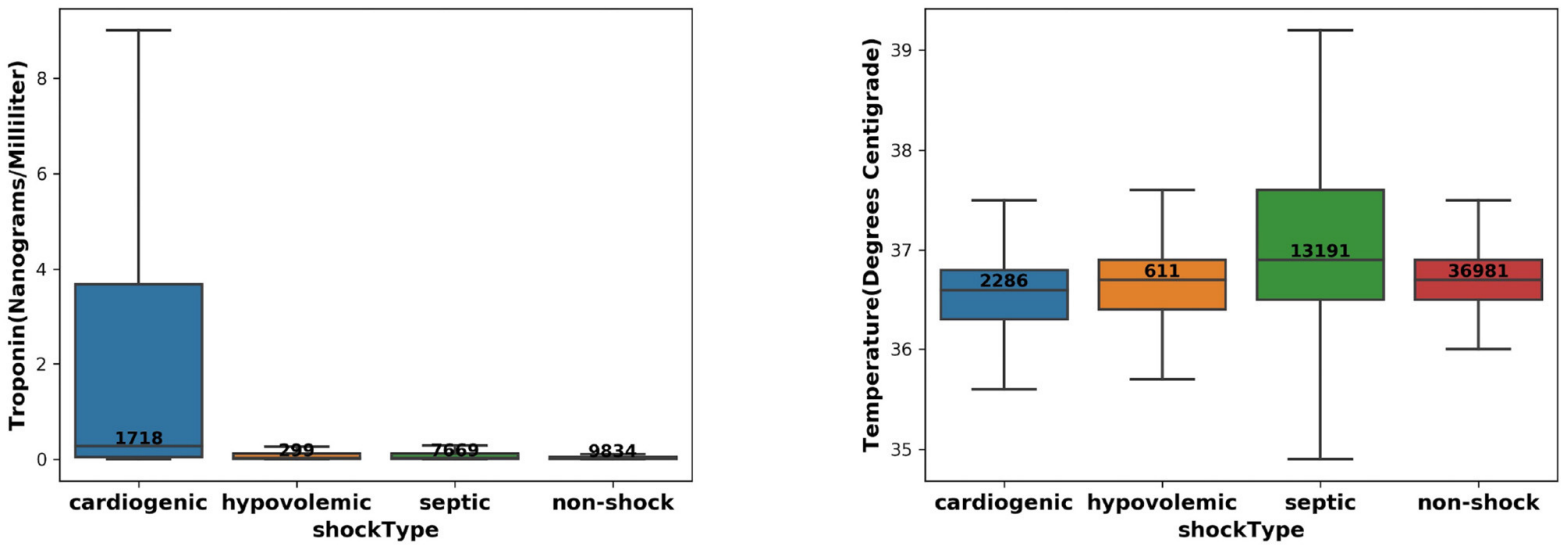
The distribution of Troponin **(left)** and Temperature **(right)** over the CS, septic/hypovolemic shock, and non-shock groups.

We took the input variables extracted at 2 h before the intervention onset and compared the distribution of Temperature and Troponin over the target and control patient groups. The results are shown in Figure 1 and show that (1) CS patients have the highest Troponin levels; (2) Septic shock patients have highest Temperature. These were consistent with pathophysiology and existing literature (10–13), which can serve as a “sanity check” on the reliability of the extracted patient cohort.

### 2.2. Model Training

Our objective was to predict the onset of CS 2 h earlier. Given that the input features were extracted from 1 to 12 h before the intervention onset for each patient, we defined the positive samples as input feature values measured at 2 and 1 h before the intervention onset time for CS patients.

The negative samples consist of the input feature values (1) measured at 3–12 h before the intervention onset for CS patients; and (2) measured at 1–12 h before the intervention onset for septic/hypovolemic shock patients and non-shock patients. In contrast, the negative samples will not develop CS within 2 h. In the design matrix used for model training, there were 8,024 samples in the positive class and 1,375,468 samples in the negative class, resulting in 0.58% prevalence for the positive class. Due to the class imbalance, we assigned higher weights to samples in the positive class to make the total weights of positive and negative class to be equal.

We randomly split all patient encounters into five-folds, where the first three-folds were used for model training, the fourth-fold for validation and the fifth-fold (hold-out test set) for model performance evaluation. For the training set, validation set and test sets, we have provided the corresponding number of unique patient encounters, number of data samples and those with positive class labels in Section 2 in the Supplementary Material.

To identify the model that can achieve both high classification performance and high interpretability, we evaluated LR and three different types of machine learning models, including XGB, multiple layer perceptron (MLP) (14), and temporal convolutional network (TCN) (15). For each model, we ran extensive hyperparameter search to maximize the AUC score on the validation set. The rationale of evaluating these models and the detailed hyperparameter search are described in Section 3 in the Supplementary Material. The optimal validation AUC corresponding of the optimal hyperparameter setting of each model is in Table 6. The XGB model consisting of 500 decision trees of depth 2 with learning rate 0.1 achieved the highest validation AUC score of 0.88.

**Table 6:** Optimal validation AUC score of each model.

**Model**	**LR**	**XGB (depth = 1)**	**XGB (depth > 1)**	**MLP**	**TCN**
Validation AUC	0.84	0.87	0.88	0.87	0.87

## 3. Results

In this section, we evaluated the model performance using multiple classification metrics. The model performance across different patient subpopulations are also presented. Furthermore, we provided interpretation tools to analyze the clinical patterns learned by the model.

### 3.1. Model Evaluation

We evaluated the model performance on the test set using the AUC, the Area Under the Precision Recall Curve (AUPRC) and the Break-Even Precision Recall (BEPR). Besides evaluating the performance on all test patients from different types of care settings, we also showed the performance on patients belonging to each care settings. It is to be noted that accurate care setting information was only available for a subset of patient encounters. Therefore, the number of patients belonging to ED, WARD, and CC were smaller than the total number of patients.

#### 3.1.1. Evaluation at All Time Steps

We first evaluated the model performance using samples collected from 12 to 1 h before the intervention onset of test patients. The results are summarized in Table 7.

**Table 7:** Model performance on all test patients as well as patients belonging to different care settings.

**Care setting**	**# Patients**	**Target prevalence (%)**	**AUC**	**AUPRC**	**BEPR**
All test patients	279,780	0.60	0.87	0.11	0.19
ED	33,684	0.41	0.84	0.11	0.20
WARD	14,292	0.42	0.97	0.14	0.22
CC	23,016	0.93	0.81	0.068	0.14

The model achieved AUC score of 0.87 on all test patients. Comparing model performance across different care settings in terms of AUC, the model performed best in WARD with AUC equal to 0.97, which was followed by ED with AUC of 0.84 and CC with AUC of 0.81. To correctly interpret the values of AUPRC and BEPR, one needs to take into account that they are negatively affected by the extremely low prevalence of the positive class (0.595%) resulting from the approach taken to define positive and negative samples (with each patient contributing 12 samples of which at least 10 are negative).

**Figure 2:**
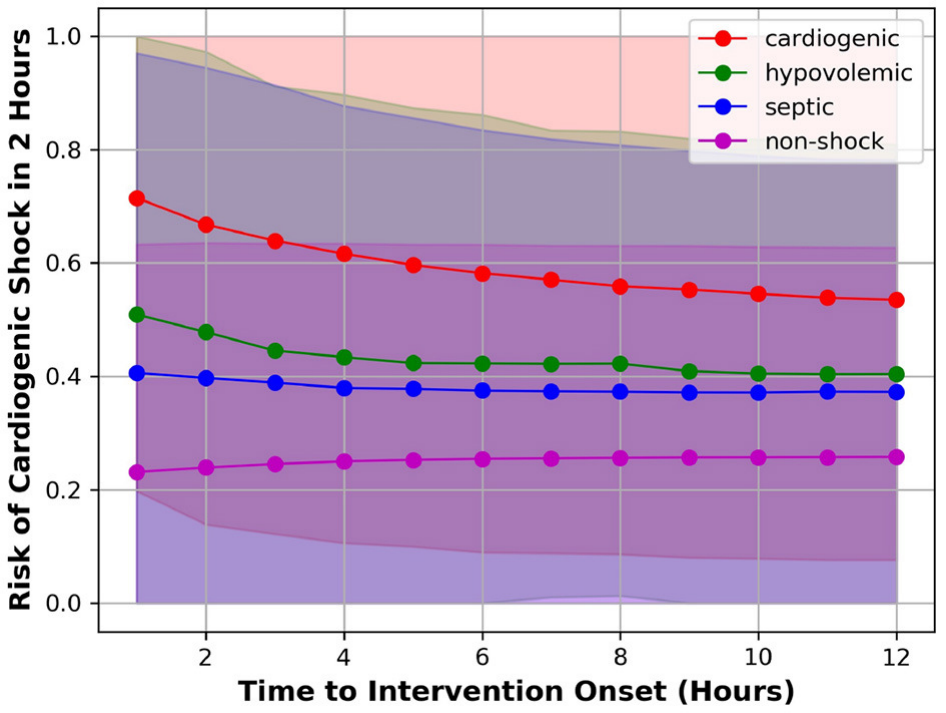
Mean risk of patient having CS (red), hypovolemic shock (green), septic shock (blue), or non-shock (magenta).

To understand how well the model can separate the positive class from the negative class at different time steps prior to intervention, we applied the model to each test patient over time and compute the risk of developing CS in 2 h. At each time point, we computed the mean and standard deviation (σ) of risk scores of patients having (1) CS; (2) hypovolemic shock; (3) septic shock; and (4) non-shock. For these four patient groups, the plot of mean risk over time is shown in Figure 2. The lower bound and the upper bound of the shaded area were computed by subtracting 2σ and adding 2σ from the mean risk (95% confidence interval). Figure 2 showed that the average risk of CS kept increasing as the time moves closer to the intervention onset, indicating the model detected higher risk of CS as patients deteriorate; Second, the average risk of developing CS in 2 h was higher for patients labeled as CS compared to patients labeled as septic/hypovolemic shock, whose average risks were further higher than no-shock patients. This means the model can discriminate between CS and non-CS patients, including septic/hypovolemic shock and non-shock. Third, the 95% confidence intervals were wide for all four patient groups, indicating highly variable risk profiles within each patient group.

#### 3.1.2. Evaluation at 2 h Before Intervention Onset

We were also interested in the model performance evaluated on samples collected at 2 h before the intervention onset. This analysis can demonstrate whether the model can correctly distinguish the onset of CS (for positive samples) from control. The results are summarized in Table 8.

**Table 8:** Model performance on all test patients as well as patients belonging to different care settings at 2 h before the intervention onset.

**Care setting**	**# Patients**	**Target prevalence (%)**	**AUC**	**AUPRC**	**BEPR**
All test patients	23,315	3.6	0.87	0.32	0.38
ED	2,807	2.5	0.85	0.15	0.12
WARD	1,191	2.5	0.98	0.67	0.60
CC	1,918	5.6	0.78	0.21	0.27

The model achieved AUC score of 0.87 on all test patients. In terms of AUC, the performance on WARD was still better than ED, which was followed by CC. Compared to the evaluation at all time steps, the values of AUPRC and BEPR were much higher due to the higher prevalence (3.6%) of the positive class (each patient contributing with one sample only, either positive or negative).

#### 3.1.3. Subpopulation Analysis

To increase clinicians' trust of the model prediction, using samples collected at 2 h before the intervention onset, we also evaluated the model performance over different patient subpopulations. The model can achieve greater clinical value if its performance is satisfactory in the subpopulation. In pursuit of this, we defined patient subpopulations using the following criteria.

1. AMI indicates the subpopulation consisting of patients with previous diagnosis of AMI within the past 1 year.
2. CHF indicates the subpopulation consisting of patients with previous diagnosis of CHF within the past 1 year.
3. ECHO indicates the subpopulation consisting of patients receiving an echocardiogram since admission.

Besides AUC, AUPRC and BEPR, we also show the recall value when selecting a decision threshold to make PPV (precision) equal to 0.5. The results are shown in Table 9.

**Table 9:** Model performance over four different subpopulations.

**Subpopulation**	**# Patients**	**Target prevalence (%)**	**AUC**	**AUPRC**	**BEPR**	**Recall@PPV = 0.5 (%)**
All	23,315	4	0.87	0.32	0.38	22
AMI	24	33	0.90	0.88	0.75	88
CHF	247	29	0.81	0.57	0.58	80
ECHO	825	15	0.89	0.60	056	68

For the analyzed patient subpopulations, the AUPRC, BEPR and Recall@PPV=0.5 values were significantly higher due to higher prevalence of the positive class. Therefore, the model should be given higher trust when applied to patients belonging to these three subpopulations.

### 3.2. Model Interpretation

**Figure 3:**
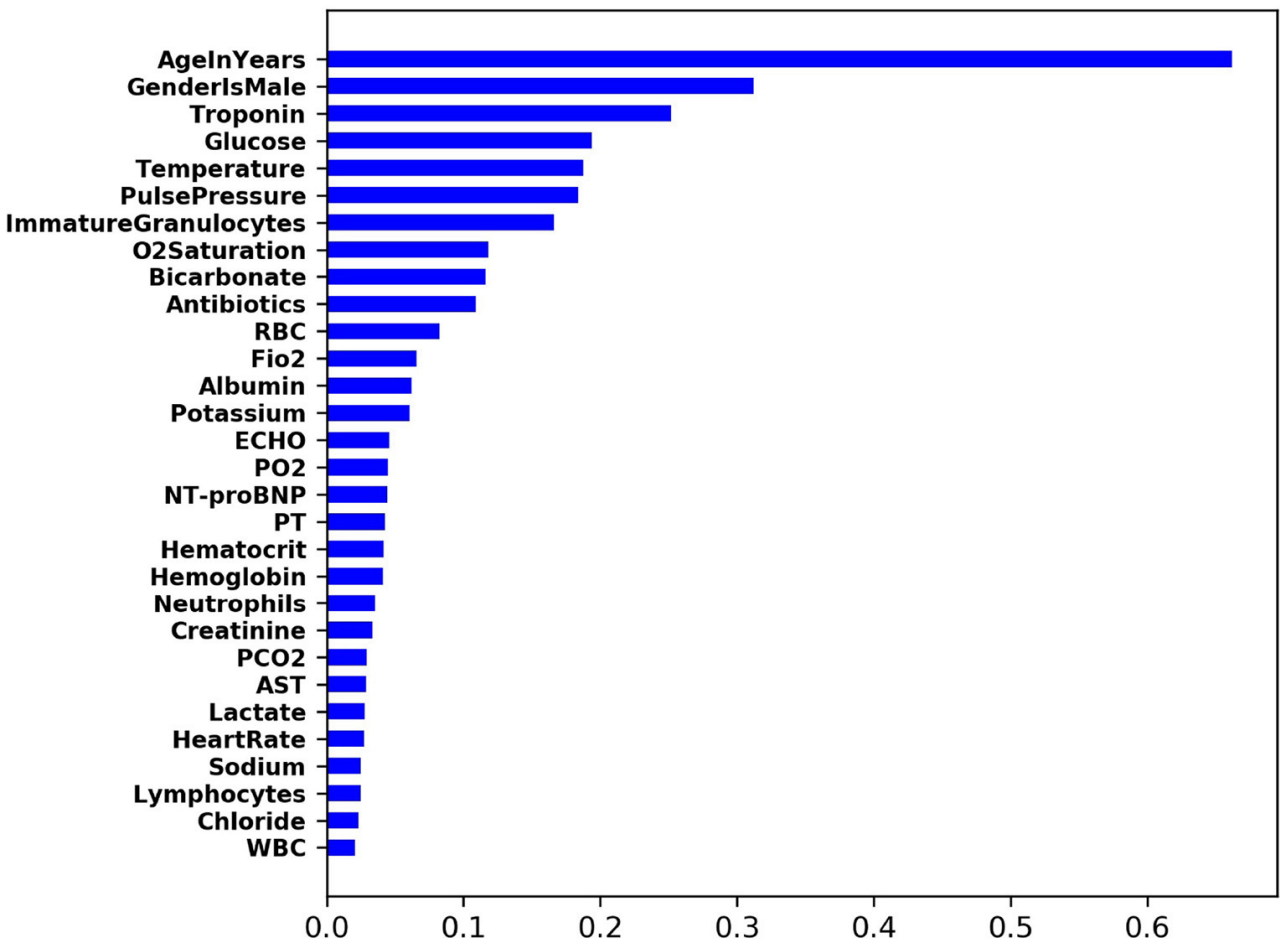
Top-ranking input features that are predictive of CS.

To understand the importance of input feature contribution in prediction of CS, we computed SHAP value (16) across all training samples. For each sample and feature pair, its SHAP value measures the importance of the feature to the prediction of the sample. Therefore, for each feature, the average of the absolute SHAP value of all training samples can be used to measure its global feature importance. Figure 3 shows the list of top-ranking input features that are predictive of the onset of cardiogenic shock in 2 h.

#### 3.2.1. Top-Ranking Risk Factors of CS

**Figure 4:**
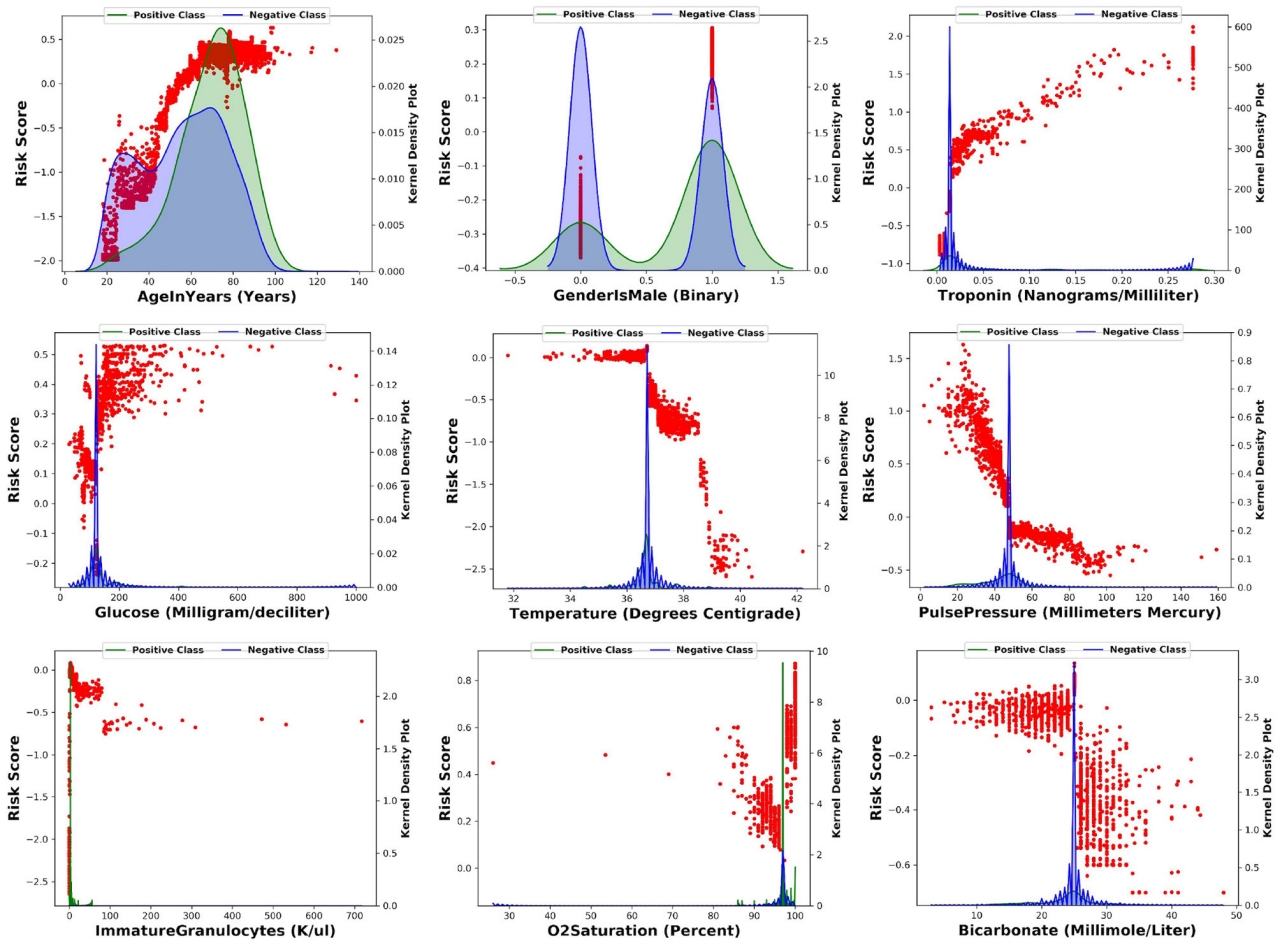
For each of the top nine most importance input features, this figure shows the scatter plot of SHAP values against the feature value.

For the top nine most important input features, we made scatter plots of SHAP values against the feature value (in red dots). This illustrates how the contribution to the model risk increases or decreases as the feature value increases. Furthermore, we also made the distribution of feature values of the positive class (in green) and negative class (in blue) in the same figure. The results are summarized in Figure 4.

The model learns the following risk factors contribute to being identified as higher risk of developing CS: (1) The risk of CS increases with respect to the patient's age (17); (2) Males have higher risk than females (17); (3) Higher troponin level is associated with higher risk of CS (12, 13); (4) Higher glucose level is associated with higher risk of CS (18); (5) Lower body temperature (cold skin) is associated with higher risk of CS (19); (6) Lower pulse pressure is associated with higher risk of CS (9); (7) Medium level of immature granulocytes (IG) is associated with higher risk of CS: high level of IG indicates infection, sepsis, and septic shock. Given about 30% of CS patients are also diagnosed with sepsis, the IG of CS patients are higher than no-shock patients; 8) Higher O2 saturation is associated with higher risk of CS possibly because CS patients receive oxygen and are often intubated; 9) Lower bicarbonate is associated with higher risk of CS (20).

These risk factors can either be supported from existing clinical literature or explained by clinical experts. Therefore, the model captures clinically meaningful patterns from the input variables to predict the onset of CS.

## 4. Discussion

We first review related works and discuss their novelties and limitations. Second, we highlight the key contributions of this work. Third, the limitations of this work are presented.

### 4.1. Related Works

Much of the currently available literature on CS prediction focuses on variables that are predictive of mortality in patients who have already developed CS (21). Factors such as renal failure, lactic acidosis, cardiac arrest, and number/doses of vasopressors are consistently associated with short-term mortality. However, by the time patients are in this situation, mortality is high irrespective of management. The need to identify these patients early is being recognized, increasingly emphasized, and is reflected in contemporary shock classification scores, such as the SCAI shock staging system (2). For example, patient in SCAI Stage A is defined as a patient at risk for developing shock whereas a SCAI Stage B patient is in beginning shock. Therapeutic interventions at these earlier stages may alter the trajectory of shock by preventing more advanced stages and importantly, cardiac arrest. However, recognizing these stages expeditiously and initiating appropriate therapy requires a trained clinician immediately at the bedside to make a subjective determination, which is not always possible, especially in smaller hospitals and during nights and weekends when staffing is less robust.

Studies on predicting the likelihood of developing CS are fewer and have predominantly focused on the acute MI population (3, 22–25). Age, baseline blood pressure, physical exam findings, laboratory values including NT-proBNP, success of revascularization and angiographic findings predict the likelihood of development of CS in AMI-CS (3). Similar parameters are implicated in non-ischemic-CS, with the exception of angiographic and revascularization data (26). There are also differences in rates of univentricular vs. biventricular shock development in ischemic and non-ischemic shock which has management and prognostic implications (27). However, these were generally retrospective analyses from registries or randomized clinical trials testing specific therapies, which may not reflect the general undifferentiated CS population. Furthermore, identified factors are often non-modifiable, not available early in the disease process, and often from eras that don't reflect contemporary management. Most importantly, these studies were also not designed with the intent to prospectively influence management in order to avoid the development of severe CS. In the current era, the ORBI study (28) developed and validated a risk score for development of CS that included 11 variables, including age >70, prior stroke/TIA, cardiac arrest, anterior STEMI, first medical contact to PCI delay > 90 mins, Killip class, heart rate >90/min, a combination of systolic blood pressure <125 and pulse pressure < 45, glycaemia >10 mmol/L, left main culprit lesion, and post-primary PCI thrombolysis in myocardial infarction flow grade < 3. The different scores could identify CS incidence ranging from 1.3 to 31.8%. Whereas, this model showed net clinical benefit compared to admission hemodynamic parameters, it was restricted to STEMI patients undergoing primary PCI, where there is already a heightened clinical suspicion for CS and patients have more intensive standard monitoring postoperatively.

An important constraint in the aforementioned studies is that predictors were derived using regression analysis methods. While regression analysis is well-suited to test associations between predictors and outcomes, it is not primarily designed to determine the likelihood of future outcomes (prediction analysis). Furthermore, regression analysis often assumes that the relationship between a predictor and outcome remains linear which may not account for various interactions of different variables on outcomes, and may have unstable effects when there are many predictors relative to number of effects (29). In contrast, machine learning models don't have a pre-specified model to fit; rather the data is evaluated for the best fit and the model is thus estimated, after which validation and testing are performed. Different machine learning methods have different advantages and disadvantages, so multiple methods are evaluated to provide the most optimal results for the individual question at hand. For this analysis, we evaluated LR as well as multiple machine learning models. XGB, a technique that can handle sparse data, allows parallel decision trees, has enhancements to avoid overfitting, models non-linear association between inputs and outputs and interactions between the inputs, provided the highest precision.

The use of machine learning in risk prediction for CS is in its infancy. Zweck et al. (30) utilized machine learning to phenotype CS using retrospectively collected data, and were able to identify and validate three distinct phenotypes with prognostic implications. Bai et al. (31) evaluated LR, least absolute shrinkage and selection operator (LASSO), support vector regression (SVM) and tree-based ensemble machine learning models (LightGBM) and XGB to predict CS risk in STEMI patients. The linear models built on LASSO and LR had the highest predictive power with AUC of 0.92. Eight predictors, including Age, CKD, WBC, Hgb, AST, LDH, Shock index, and delay to first medical contact > 12 h, were predictive of development of in-hospital CS. The specific timing of CS development was not evaluated. Rahman et al. (32) utilized machine learning methods in an attempt to identify a cohort of patients at higher risk of developing CS from a population of patients admitted to three hospitals with acute decompensated heart failure. A novel feature of this study is continuous monitoring of EHR data. The high-risk cohort had 10.2 times higher prevalence of developing CS within the next 24 h compared to the low-risk group, although the overall positive predictive value of the model at various thresholds was under 10%.

### 4.2. Contributions

Our results substantially expand on the currently available methods. First, the model is not restricted to a subpopulation of patients (e.g., post-STEMI or ADHF), but rather evaluates a general population of hospitalized patients who are at risk for CS development. Second, the model was derived with data from a large regional healthcare system with community hospitals, mid-level hospitals, and tertiary academic hospitals. This limits selection bias and allows generalizability. Third, the model is designed to continuously monitor EHR data rather than using a single snapshot of clinical variables, to provide real time actionable data. The 2-h time frame for earlier prediction allows notification and time for clinicians to immediate mobilize necessary resources for more advanced interventions during working and off hours, and allow time to arrange transfer to higher levels of care if necessary. Fourth, the model can be utilized in various care locations, such as ED, wards, or CC, with highest discrimination in general hospital wards where the level of intensive physical monitoring is lowest and risk of delays in treatment the highest. Finally, subpopulation analysis, such as those with prior AMI or CHF shows varying discriminatory capacity, which may be utilized in targeting disease-specific therapies.

The input features that had the most impact in predicting CS were, in decreasing order, age, male gender, troponin levels, glucose levels, temperature, pulse pressure, and immature granulocytes. These demographic variables or examination and laboratory values are routinely measured in clinical care and don't require additional resources, yet were powerful predictors of CS. Algorithms such as these may provide an opportunity for earlier recognition of shock and facilitate management, particularly in populations who have delayed recognition and poorer outcomes with current standard practices. Variables such as lactate and creatinine which are validated predictors of outcomes after CS had lower impact, likely because patients are detected prior to significant end-organ hypoperfusion and dysfunction. Importantly, the model could provide meaningful discrimination between cardiogenic and septic shock. Often cardiogenic shock misdiagnosed as septic shock leads to a series of management decisions (e.g., intravenous fluids) which have the potential to worsen CS and create additional problems such as need for intubation. Predictive models such as these could heighten early suspicion of CS, particularly in ED or units where septic shock is much more commonly observed and clinical management of hypotensive patients is geared toward sepsis protocols.

Practical applications of models such as these could be varied, depending on clinical situation. It can be implemented in a dashboard in the patient chart without a specific alert. Other models, such as the sequential organ failure assessment score (SOFA score) are already routinely implemented in modern EHRs in this manner. It can be implemented as an electronic alert in the EMR at a certain threshold with the decision for further interventions left at the discretion of the provider. Alternatively, more active interventions, such as activation of rapid response teams or shock team alert could also be automatically triggered. In an individual patient, progressively more aggressive measures could also be activated depending on timing and risk of developing CS. It is important to have reliable models that meaningfully impact diagnosis and treatment with reasonable likelihood but are not too repetitive in order to avoid situations where clinicians get notification fatigue and disregard the alerts.

### 4.3. Limitations

We did not individually adjudicate CS events but rather the diagnosis was based on ICD codes. This may have decreased sensitivity and/or specificity but likely incorporated events that the treating clinicians deemed relevant. We mitigate this by using CS-specific interventions to make CS labels more accurate. Another limitation was the inability to extract some data that we deem relevant predictors. For example, left ventricular ejection fraction measurements were recorded as a free-text value in a separate echocardiographic reading software which is then imported as PDF document into the primary EHR and is not extractable. Similar limitations were present for angiographic and hemodynamic data. Integration of various EHR platforms and uniform recording methods may expand utility of EHR data. Artificial intelligence methods to extract relevant values were in progress but were not available for this analysis. Third, the use of first intervention timing as opposed to a blood pressure threshold to delineate CS onset has limitations. However, as increasingly recognized by the SCAI shock classification and others, CS occurs on a continuum rather than at a specific discrete BP threshold, and the ancillary findings in addition to BP that used in prospective clinical trials to diagnose cardiogenic shock, such as physical findings of cool extremities or altered sensorium, or hourly urine output at onset of shock, are not readily available from retrospective EMR records. Implantation of MCS devices takes time to arrange, and a patient may, in some cases, have already been diagnosed with CS 2 h prior to the initiation of MCS. However, the vast majority of patients had inotropes/vasopressors which are instituted rapidly after diagnosis rather than a MCS device as the first intervention. Fourth, the time to CS onset and the time to intervention may differ across different institutions and changing institutional practice protocols over time. Finally, this model was derived from retrospectively obtained data used for clinical purposes. Prospectively collected data (e.g., continuous hemodynamic monitoring, serial lactate) may improve precision for CS prediction but is not widely practical in routine clinical care.

## Data Availability Statement

The datasets presented in this article are not readily available because this dataset is owned by Banner Health. Requests to access the datasets should be directed to yale.chang@philips.com.

## Ethics Statement

The studies involving human participants were reviewed and approved by the Banner Health Institutional Review Board. Written informed consent for participation was not required for this study in accordance with the national legislation and the institutional requirements.

## Author Contributions

CA and DA proposed the problem setup. FV, LW, PT, and DS worked on obtaining the IRB approval of data collection. YC, CA, SR, JD, ML, and DA worked together to extract and analyze the patient data. All authors participated in the discussion of the results. All authors contributed to the article and approved the submitted version.

## Funding

This work was supported by both Philips Research North America and Banner Health.

## Conflict of Interest

YC, SR, JD, FV, and LW are the employee of Philips Research. Philips is a healthcare company that manufactures medical devices. The remaining authors declare that the research was conducted in the absence of any commercial or financial relationships that could be construed as a potential conflict of interest.

## Publisher's Note

All claims expressed in this article are solely those of the authors and do not necessarily represent those of their affiliated organizations, or those of the publisher, the editors and the reviewers. Any product that may be evaluated in this article, or claim that may be made by its manufacturer, is not guaranteed or endorsed by the publisher.
