# Supplementary material for: Early Prediction of Cardiogenic Shock Using Machine Learning
Source: Front Cardiovasc Med. 2022 Jul 13;9:862424. doi: 10.3389/fcvm.2022.862424 (PMC9326048; doi:10.3389/fcvm.2022.862424)
Supplement: Supplementary file 1 [file Data_Sheet_1.PDF]

Supplementary Material

1 INPUT FEATURE DESCRIPTIONS

Table S1 explains the meaning of a subset of input variables to the model.

1.1 Mapping Conventional Troponin to High-Sensitivity Troponin

From 2010 to 2017, most troponin measurements utilized the conventional troponin assay. Since 2018, high-sensitivity troponin use was implemented. To train a model that can take high-sensitivity troponin as input, we learn a mapping between conventional troponin and high-sensitivity troponin by extracting high-sensitivity troponin measurements from the same set of hospitals from 2019 to 2020. After learning the mapping, we convert the conventional troponin measured from 2010 to 2017 to high sensitivity troponin.

2 SAMPLE SIZE OF TRAIN, VALIDATION, AND TEST SET

Table S2 shows the sample sizes of the train, validation and test set.

Table S1. This table explains the meaning of a subset of input variables to the model

ALT	Alanine Aminotransferase
AMI	1 if the patient had diagnosis of AMI within the past one year and 0 otherwise
AST	Aspartate Aminotransferase
AVPU_Scale	“Alert, verbal, pain, unresponsive” scale measure a patient’s level of consciousness
Antibiotics	1 if the patient receives antibiotics in the last 48 hours and 0 otherwise
BUN	Blood Urea Nitrogen
BloodCulture	1 if the patient was taken blood culture in the last 48 hours and 0 otherwise
Cardiomyopathy	1 if the patient had diagnosis of cardiomyopathy within the past one year and 0 otherwise
CKMB	Creatinine Kinase-MB
CKMB_CKTotal	The ratio between Creatinine Kinase-MB and Creatinine Kinase-Total
CKTotal	Creatinine Kinase-Total
CRP	C-Reactive Protein
CVP	Central Venous Pressure
ECHO	1 if the patient ever received ECHO in the current stay
EWS_CNS	EWS CNS Level of Consciousness
GFR	Glomerular Filtration Rate
INR	International Normalized Ratio
MitralRegurgitation	1 if the patient had diagnosis of Mitral Regurgitation within the past one year and 0 otherwise
PAWP	Pulmonary Artery Wedge Pressure
PEEP	Positive End-Expiratory Pressure
PIP	Peak Inspiratory Pressure
PT	Prothrombin Time
PTT	Partial Thromboplastin Time
RBC	Red Blood Cell Count
SAFE_SIRS	1 if two SIRS criteria and one OD criteria were met within the last 48 hours(Khurana et al., 2016)
TroponinDelta	The difference between the maximal and the minimum troponin measured since the patient’s admission
WBC	White Blood Cell Count

Table S2. This table shows the number of unique patient encounters, number of data samples and those with positive class labels for training, validation, and test set.

	# unique patients	# data samples	# positive data samples
Train set	68,852	826,224	4766
Validation set	23,124	277,488	1592
Test set	23,315	279,780	1666

Table S3. This table compares the properties of different machine learning models

Model Name	Non-linearity	Feature Interaction	Temporal trends	Interpretability
LR				YES
XGB (depth=1)	YES			YES
XGB (depth>1)	YES	YES		SHAP
MLP	YES	YES		SHAP
TCN	YES	YES	YES	NO

Table S4. this table summarizes the hyperparameter search range the optimal hyperparameter of each model.

Model Name	Hyperparameter Search Range	Optimal Hyperparameters
LR	L2 regularization coefficient: {10^-9,...10^4}	0.0001
XGB(depth=1)	learning rate: {0.0001,0.001,0.01,0.1,0.2,0.3}<br>number of trees: {100,200,500,1000}	learning rate=0.2<br>number of trees = 1000
XGB(depth>1)	tree depth: {2, 3, 4, 5}<br>learning rate and number of trees:<br>same as above	tree depth = 2<br>learning rate = 0.1<br>number of trees = 500
MLP	number of hidden layers: 1 - 10<br>dimension of hidden layers:<br>{10,20,50,100,200,500,1000}<br>L2 regularization coefficient:<br>{10^-4, ..., 10^4}	number of hidden layers = 10<br>dimension of hidden layers = 1000<br>L2 regularization coefficient = 10^-4
TCN	number of hidden layers: 1<br>dimension of hidden layers:<br>{10,20,50,100,200,500}<br>L2 regularization coefficient: {10^-6, ..., 10^2}	number of hidden layers = 1<br>dimension of hidden layers = 200<br>L2 regularization coefficient = 10^-4

3 HYPERPARAMETER SEARCH OF XGBOOST, LOGISTIC REGRESSION, MULTIPLE LAYER PERCEPTRON AND TEMPORAL CONVOLUTIONAL NETWORK

We evaluate XGBoost, Logistic Regression, Multiple Layer Perceptron and Temporal Convolutional Network. We compare the properties of these models in terms of whether they can 1) capture non-linear relationship between input and labels; 2) handle interactions between input features; 3) incorporate temporal trends of input features; and 4) be interpreted by users. The comparison results are summarized in Table S3. For models that can't naturally incorporate interactions or temporal trends of input features, feature engineering can be applied to manually define feature interaction terms or temporal feature statistics. However, these approaches involve significant manual efforts, so we don't consider them in this work.

We search for the optimal hyperparameter of each model and summarize the results in Table S4.

REFERENCES

Khurana HS, Groves Jr RH, Simons MP, Martin M, Stoffer B, Kou S, et al. Real-time automated sampling of electronic medical records predicts hospital mortality. *The American journal of medicine* **129** (2016) 688–698.
